# The Sentinel Lymph Node in Breast Cancer: Problems Posed by Examination During Surgery. A Review of Current Literature and Management

**DOI:** 10.3389/fsurg.2018.00056

**Published:** 2018-11-14

**Authors:** Jean Bouquet de Jolinière, A. Major, F. Khomsi, N. Ben Ali, L. Guillou, A. Feki

**Affiliations:** ^1^Department of Gynecology Obstetrics, Cantonal Hospital, HFR, Fribourg, Switzerland; ^2^Argotlab and Synlab Laboratories, Department of Pathology, Lausanne, Switzerland

**Keywords:** sentinel node, examination during operation, breast cancer, immuno-histo chemistry, axillary curage

## Abstract

The presence of tumor cells can be identified in the lymph node when metastasis has occurred from the primary cancer site into the lymph node (1) If the sentinel lymph node ganglion is negative for the presence of tumor cells at the time of histological examination, the other lymph nodes are also negative in 99% of cases. If no tumor cells are identified in the sentinel lymph node ganglion by histological examination, the other lymph nodes are also negative for the presence of tumor cells in 99% of cases. The sentinel lymph node advantageously replaces axillary dissection as a staging method in breast cancer T1 and T2 (2). Approximately 40% of breast cancers metastasize to axillary lymph nodes and metastatic extension depends on disease stage. Sentinel lymph nodes are affected in the following stages: T1a (4.3%), T1b (19.5%), T1c (23.8%), T2 (48.9%), T3 (66.7%).

## Introduction

In recent years the management of breast cancer has changed considerably. One such example of this is the disappearance of *great axillary curage*. The study of lymph nodes has a prognostic value and guides decision making for further treatment (3–6). Examination of the sentinel lymph node has become the gold standard. Before performing axillary clearing, evidence of metastatic invasion of at least three lymph nodes is recommended. The examination of the sentinel node during surgery remains arbitrary as micro-metastases cannot be detected. The sensitivity of this technique is dependent on the sections obtained, and interpretation can be challenging for pathologists. Oftentimes, further examination in the laboratory is necessary to *obtain and validate reliable* results.

Sections are conditioning this examination and many pathologists and many pathologists don't want to take this responsibility. Only the laboratory examination reassures the results.

The purpose of the present article is to demonstrate how and why through a review of the current literature.

*Two* techniques are currently available: standard and advanced. The present article *provides a review of the current literature*, and discusses the advantages and disadvantages of examination during surgery of the sentinel node (7–12).

## Standard histological analysis of the sentinel node

### Analysis of the sentinel node ganglion have revolutionized breast cancer surgery

- Aims: detect macro metastases (>2 mm), micro metastases (0.2–2 mm). Isolated tumor cells or clusters ≤ 0.2 mm are often only detected by immunohistochemistry.
- *Technical*:
  - *Imperative*: cut the ganglion every 2 mm, either vertically (small ganglion ≤ 4 mm) or transversely (ganglion> 4 mm).
  - Recommended: 3 depths (minimum).
- Results: All macroscopic and micro metastases are detected.

### A brief history of sentinel node examination

Surgery has always used minimally invasive techniques in order to limit associated morbidities and facilitate post-operative surgical *recovery*. The examination of sentinel nodes was first performed in other types of cancer, before being applied to breast cancer. Below are some examples of this:

- 1977: The sentinel node technique was developed by R.M. Cabanas to evaluate nodal extension in penile cancers.
- 1992: The sentinel node technique was applied to melanoma:
- 1993: The sentinel node technique was applied to breast cancer.
- 2013: 20 years later, there is still no standardized and universally accepted protocol for anatomo-pathology analysis of the sentinel node. But, fortunately, there are recommendations and guidelines.

### Standard analysis techniques for the ganglion examination in classical pathology: (13)

There are some rules to follow that demonstrate how an extemporaneous examination can be arbitrary. In a standard analysis, the different sections used make it possible to examine the sentinel lymph nodes *perfectly*, without omitting the minimal *attacks* or even isolated cells. All macro and micro metastases are detected. It is necessary to make deep cuts (at least 3) on each. *It is necessary to obtain thick sections*.

*It is then possible to evaluate:* The size of the macro metastases are noted in mm and the distance to the capsule, the presence or absence of a capsular rupture, the number and size of micro metastases and their locations, at last the presence or absence of isolated tumor cells (IHC) (14–16). As previously described, each section of lymph node should not exceed 2 mm in thickness.

On each block, do make at least three deep cuts to avoid missing metastasis. On the diagrams, in red are the metastases and the lines correspond to the cuts made. The lymph node thickness is important to consider as it is the reason why the serial cuts are important (*Schema Images* 1, 2, 3).

### Meaning of the presence of tumor in the sentinel lymph node

The presence of tumor in the sentinel lymph node must be considered *according to* different stages. In case of:

- Macro metastases (picture 5): There is a high risk of axillary residual disease (other positive lymph nodes frequently observed).
- Micro metastases (picture 4): There is an axillary residual disease in 10% of patients.
- Isolated tumor cells (picture 6): Rarely associated with an axillary residual disease and have no proven prognostic significance.

These slides show how difficult and random it is to carry out in both cases an examination during surgery.

This is why an immuno-histochemical analysis makes the diagnosis more reliable. Thus, it will be the final analysis of the node that will allow a finer diagnosis and the discovery of isolated tumor cells. While this result may not affect future treatment, it has the advantage of avoiding diagnostic errors.

## Sentinel lymph node: advanced histological analysis

### Immunohistochemistry

This examination facilitates the detection of isolated micro metastases and tumor cells (Ac anti cytokeratin), however false positives may be observed (benign epithelial inclusions, degenerative cells in transit, keratin-positive dendritic cells, epidermal cells in transit, etc.).

*This is why the* technique is become facultative according to current American clinical .society of pathology recommendations.

### Molecular biology

Technique intended for the detection of isolated tumor cells. This technique is not performed routinely. The identification of certain molecular subtypes of cancers (luminal A and luminal B), in a positive sentinel lymph node, would be associated with a higher risk of axillary residual disease and would lead to complementary lymph node dissection (17).

## Surgical technique of sentinel node analysis

The currently surgical technique of sentinel node analysis is globally accepted (18–24). The patient receives an injection of colloid (TC 99M) the day before or the morning of the procedure and undergoes a lympho-scintigraphy. A *card* is given to the surgeon for the result.

On the day of surgery, at the beginning of the operation, the patient is anesthetized, a mini dose of blue patent is injected in peri-areolar and subcutaneously? A mini incision is made and the surgeon with a probe reads the radio activity, and looks for the blue of the ganglion (picture 9), which is removed and checked with the probe (picture 10). A noise of radioactivity is given and the piece is sent to the pathologist for the continuation of the examination.

However, it is necessary to discuss the differences between the histological and cytological analysis of a lymph sentinel node during surgery, as the *results can be discordant* (25).

## Histological analysis (per-operative) of sentinel lymph node

### Conventional indications (currently questioned)

The *challenge* is how identifying the location of extemporaneous examination of the sentinel lymph node in breast cancer *given that:*

- Detect macro metastases (>2 mm) to perform complementary axillary dissection at the same operative stage (but macro metastases can be seen in radiology (CT/MRI) and punctured/biopsied before the operation).
- Detected micro metastases (but the presence of micro metastases is no longer a formal indication for complementary axillary lymph node dissection)
- Isolated tumor cells are not taken into account.

### Technical consider another word choice like technique, or method

Histologically, it is necessary to cut the lymph node every 2 mm and examine all sections extemporaneously. Particular attention must be paid to avoid using all material and allow for proper standard histological examination after fixation.

It is also necessary carrying out cytological fingerprints.

### Results and benefits

This type of examination allows either a reliable diagnosis (90% of the cases) that conditions the practice of a complementary axillary clearing. There is a variable false negative rate (0–48%), inversely proportional to the number of deep cuts performed. The reliability through cytological fingerprinting is increased. *Sometimes, an immuno-histo-chemical examination can be performed*. But a lengthy and difficult reading is necessary as there are many artifacts due to frozen tissue: This examination requires an experienced and specialized pathologist, who is not always accessible. Lastly, the permanent loss of part of tissue for standard histological diagnosis is a risk as it may *distort* the final review.

## Cytological analysis (per-operative) of sentinel lymph node

This procedure has satisfactory specificity. Several advantages and disadvantages are described below (26, 27):

### Advantages

- It allows a reliable diagnosis in 95% of the cases that condition complementary axillary dissection (28).
- The false negative rate is 5–10%.
- Rapid immuno-histochemical examination is possible (rare).
- This technique is simple and cheap.
- Importantly, there is no tissue loss, unlike histological examination.

### Disadvantages

- Reading the tissues histologically during the operation is too long.
- This examination requires experience and training of the pathologist.
- Size and location of metastatic site not *to be* specified.
- Differentiation between macro and micro metastases is not possible.

## Discussion and literature review

Knowledge of sentinel lymph node status remains the most powerful prognostic factor in defining the adjuvant strategy of the majority of solid tumors (29).

The difficulty of the methods described demonstrates the necessity of having an experienced pathologist to *evaluate* extemporaneously micro metastases or isolated metastasis cells. As proposed by the American Society of Breast Surgeons, accreditation should be given to surgeons practicing sentinel node *examination* in breast cancer after teaching and evaluating their technique.

The axillary dissection gives only a prognostic indication (30). The tumors being diagnosed smaller and smaller, the technique of the GS has taken its place. The adjuvant therapeutic strategies depend on its results.

Indeed, the presence of isolated tumor cells has no predictive value for residual axillary disease. No pejorative prognostic value either the presence of micro metastases is associated in 10–12% of cases with residual axillary disease. No significant prognostic difference in terms of overall survival and survival without recurrence (Acozog 2011). Therefore no complementary axillary clearing is use full (28).

At the Lyon South Hospital Center (29), one study evaluated the value of lymph node and axillary sentinel node (*GS)* biopsy in 243 invasive breast cancers with non-palpable lymph nodes, according to the colorimetric (Evans blue and patent blue) and combined (colorimetric + technetium 99 m colloid) techniques. The sentinel lymph node (GS) detection rate was 225/243 (92.59%), with the colorimetric method of 89.94% and the combined method of 100%. The false negative rate was less than 2%.

In the literature, it is interesting to note that no *per*- unsure of meaning of per operative examination has been carried out to date.

The incidence of ganglion metastasis was studied in the GS in multiple-section HPS staining. If this examination was negative, the GS was examined using immunohistochemistry. The other axillary lymph nodes were examined in HPS on two sections. They are fixed in Bouin's liquid. After 24 h, they were cut into sections of 3 mm after having been included in paraffin and stained with hematoxylin phloxin saffron (HPS). If no metastasis was found in HPS, immunohistochemistry (IHC) was performed with cytokeratin KL1 (1/100 Immuno-tech dilution) on 4 mm thick sections fixed with the immuno-peroxidase method amplified by the streptavidin complex/Biotone and revealed by diaminobenzidine (Kit L. Sab. Dako®, Denmark)(29).

There must be not only a standardization of the technique of lymphatic marking, but also of the anatomo -pathological study of the GS, in particular by immunohistochemistry. It should be emphasized that for specialized teams, whatever the technique used, the results obtained are very similar with detection rates of GS>90%.

In their text, the literature is explicit. The false negative rate (FN) is certainly the most important element to consider for this diagnostic test (number of axillary adenopathies p N1 when the GS is p N0/total p N1). For the American Society of Breast Surgeons for a GS rate of 85%, the acceptable FN rate is ≤ 5% (40). In their study, the rate of FN is 7/92 (7.60%), *but if FN is removed macroscopically obviously malignant for the surgeon where the detection of the GS no longer has interest, the rate of FN Is acceptable (1/91–1.09%) -Unsure of meaning here*. Currently, the FN rate varies from one team to another, but it must be emphasized that in trained teams it is less than 10% (11, 24, 31). Using the colorimetric method, Giuliano has a FN rate of 0% (31). Miltemburg of the 1,335 cases with a GS identification rate of 83.6%, have an FN rate of 5.1% (30). In a study by Viale et al., the negative predictive value was 90.3 to 100% with a concordance between GS histology and axillary node status from 97.1 to 100% (45). These figures are found by Albertini (32).

By multiplying the sections on the axillary lymph nodes (multiple serial sections) compared to a conventional method, the rate of p N1 is increased by an average of 9% (from 4 to more than 20%). By an immuno-histo-chemical study, the p N1 level due to micro metastases increases by an average of 20% (8 to more than 40%). This is why we are not in favor of the intraoperative examination of the GS. This examination may result in loss of material and may not permit an accurate immuno-histochemical study. The extemporaneous examination of the GS only seems justified if the surgeon suspects metastasis due to macroscopic examination of this ganglion (27). The false negative rate of the extemporaneous examination of the GS is 18% for Hill et al., 17% for Veronesi (11) and 13% for Van Diest (33). False negatives are due to micro metastases detected only by serial ganglion sections or by immunohistochemistry.

In their study, the total number of micro metastases was 23/85 p N1 sentinel lymph node, with a detection rate of only 9% in IHC. For Giuliano, the micro metastases axillary level was 7.7%, 9% for Lineham et al., 10.6%, for Cox (34) and 17% for Veronesi (11, 31). In a study by Dowlasthahi, this rate was very high for small tumors as it reached 58% (35). But 50% of micro metastatic p N1 is invaded by less than ten malignant cells (34). Thus, by extensive GS study by immunohistochemistry, the number of patients with stage II cancer is increased compared with conventional histopathological examination. It is therefore necessary to remove the current controversy over the prognostic value of these micro metastases detected by immunohistochemistry and molecular biology in order to define an appropriate strategy (34–37).

In our study, the risk of leaving invaded lymph nodes in the armpit would be 35/85 (41.17%). For Veronesi (11) it would be 62 and 43% for Albertini (32).The greater the size of the micro metastases in the sentinel lymph node (GS), the greater the risk of non-GS lymph node involvement (28). It is therefore necessary to define a maximum size of micro metastases for which the risk of p N1 outside the GS is zero and does not require an axillary dissection.

## Conclusion

It is difficult to accurately identify micro metastases in the breast sentinel lymph node by extemporaneous examination. Therefore, the authors of the present article avoid performing it.

Should we continue to do extemporaneous examinations on sentinel lymph nodes?

Is it necessary to perform a lymph node dissection in the presence of micro metastases?

According to updated international guidelines, 3 invaded lymph nodes are required to perform an axillary clearing. Lastly, there is a current discussion regarding the ablation under local anesthesia of the GS at the same time as diagnostic biopsy in order to allow multidisciplinary teams to take an *ad hoc* decision and avoid two surgical interventions.

## Author contributions

All authors listed have made a substantial, direct and intellectual contribution to the work, and approved it for publication.

### Conflict of interest statement

The authors declare that the research was conducted in the absence of any commercial or financial relationships that could be construed as a potential conflict of interest. The reviewer LL and the handling editor declared their shared affiliation at the time of the review.
